# Oesophageal perforation secondary to denture ingestion requiring thoracotomy and T-tube placement: a rare case report

**DOI:** 10.1093/jscr/rjag950

**Published:** 2026-10-27

**Authors:** Konstantinos Baronos, Seohyun Lee, Sara Arshad, Marios Alogakos, Rami Ayoub, Christopher Sutton, David Bowrey

**Affiliations:** Department of General Surgery, University Hospitals of Leicester NHS Trust, Infirmary Square, Leicester, LE1 5WW, United Kingdom; Department of General Surgery, University Hospitals of Leicester NHS Trust, Infirmary Square, Leicester, LE1 5WW, United Kingdom; Department of General Surgery, University Hospitals of Leicester NHS Trust, Infirmary Square, Leicester, LE1 5WW, United Kingdom; Department of Surgery, Indiana University, Emerson Hall, 545 Barnhill Drive, Indianapolis, IN, 46202, United States; Department of Hepatopancreatobiliary Surgery, University Hospitals of Leicester NHS Trust, Groby Rd, Leicester, LE3 9QP, United Kingdom; Hepatopancreatobiliary Surgery–General Surgery Department, Al-Balqa Applied University, As-Salt, 19117, Jordan; Department of General and Upper Gastrointestinal Surgery, University Hospitals of Leicester NHS Trust, Infirmary Square, Leicester, LE1 5WW, United Kingdom; Department of General and Upper Gastrointestinal Surgery, University Hospitals of Leicester NHS Trust, Infirmary Square, Leicester, LE1 5WW, United Kingdom

**Keywords:** oesophageal perforation, foreign body ingestion, T-tube placement

## Abstract

Oesophageal perforation from foreign body ingestion is a life-threatening emergency. Dentures pose particular risk due to irregular shape, size, and radiolucency. We present a 69-year-old female who accidentally ingested a denture. Radiography identified the foreign body at C4/C5. Endoscopic retrieval failed due to mucosal embedding, and a through-and-through oesophageal tear was confirmed. She underwent emergency right thoracotomy with denture retrieval. Given localized inflammation, a 4 cm perforation, and pneumomediastinum, a T-tube was placed across the defect instead of primary repair. Post-operatively, she required intensive care, enteral feeding, and prolonged chest drainage. Recovery was complicated by suspected mediastinitis, candidiasis, and deconditioning, but she ultimately tolerated oral intake and was discharged. In complex thoracic OP with inflammation or a large defect, T-tube placement offers a reliable, oesophagus-preserving alternative to primary repair. This case highlights the diagnostic challenges of radiolucent dentures and the importance of multidisciplinary decision-making.

## Introduction

Oesophageal perforation (OP) is an uncommon but life-threatening condition, associated with substantial morbidity and mortality, particularly when diagnosis and treatment are delayed [1, 2]. While most cases are iatrogenic, foreign body ingestion remains a well-recognized cause [1, 3]. Dentures represent a particularly hazardous subset due to their large size, irregular contours, and radiolucency [3–5]. Delayed recognition of OP may rapidly result in mediastinitis, sepsis, and multi-organ dysfunction, with mortality rates significantly increasing when treatment is initiated more than 24 h after perforation [2, 6]. Management strategies are influenced by the location and extent of perforation, degree of mediastinal contamination, and timing of presentation. While contained OPs may be amenable to conservative or endoscopic management, delayed or extensive thoracic perforations frequently require operative intervention [6, 7]. Surgical options include primary repair, diversion procedures, oesophagectomy, or controlled fistulization using T-tube placement [6, 7].

## Case presentation

A 69-year-old female with type 2 diabetes mellitus, hypertension, severe asthma, and ischaemic heart disease presented following accidental ingestion of part of a denture. On admission, they had severe sharp, stabbing chest pain localized just above the sternal notch, alongside odynophagia. They were otherwise alert, hemodynamically stable and vital signs stable. Initial cervical and chest plain radiography demonstrated evidence of a foreign body fragment at C4/C5 level (Fig. 1).

**Figure 1:**
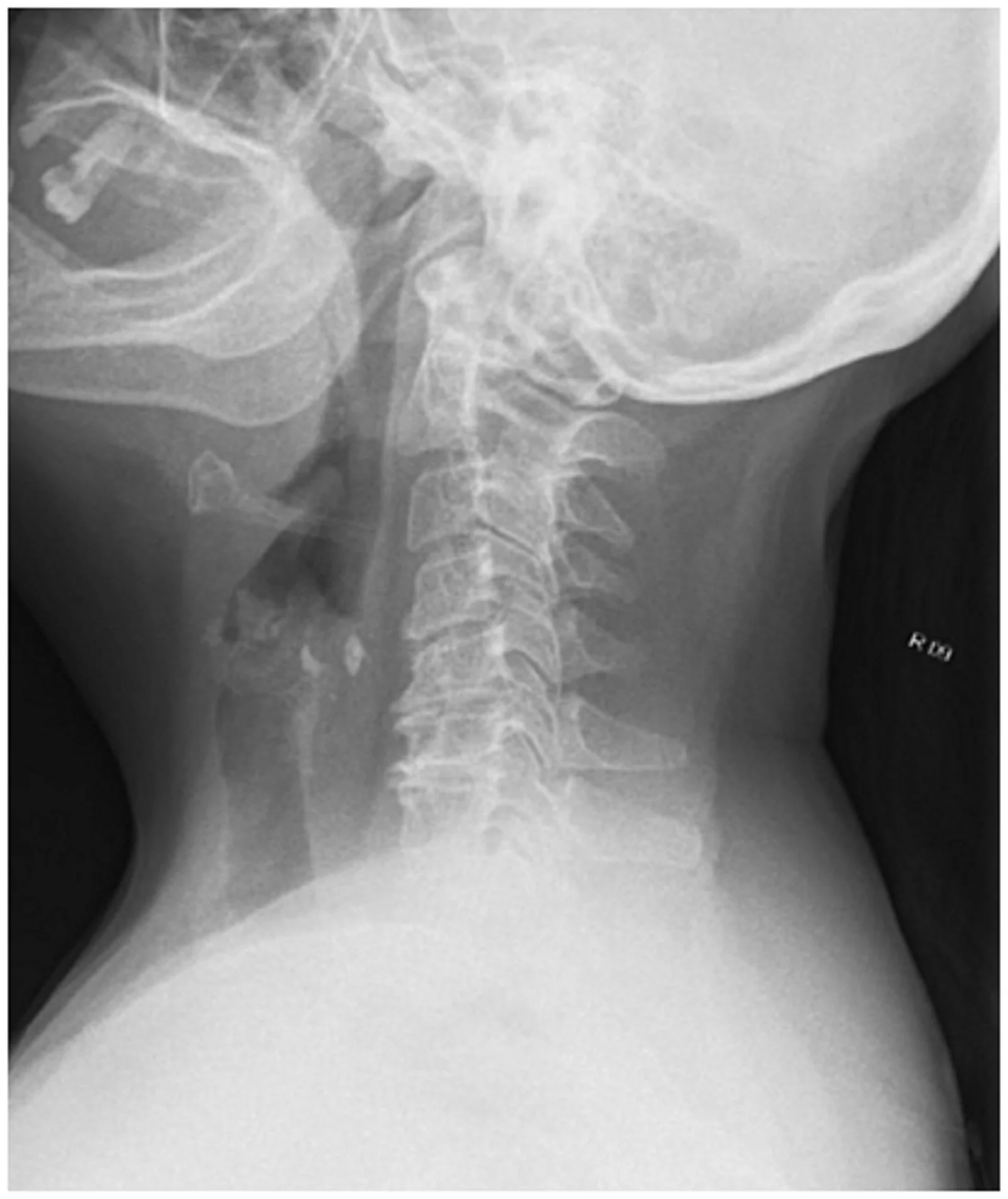
Lateral plain radiograph of the neck on the day of admission, demonstrating a radiolucent foreign body at the C4/C5 level (indicated by the arrow).

Rigid oesophagoscopy demonstrated a foreign body lodged within the upper thoracic oesophagus (Fig. 2). Attempts at endoscopic retrieval were unsuccessful due to mucosal embedding, raising concern for transmural migration, and the procedure was abandoned. The patient proceeded to emergency right lateral thoracotomy within 18 h of ingestion.

**Figure 2:**
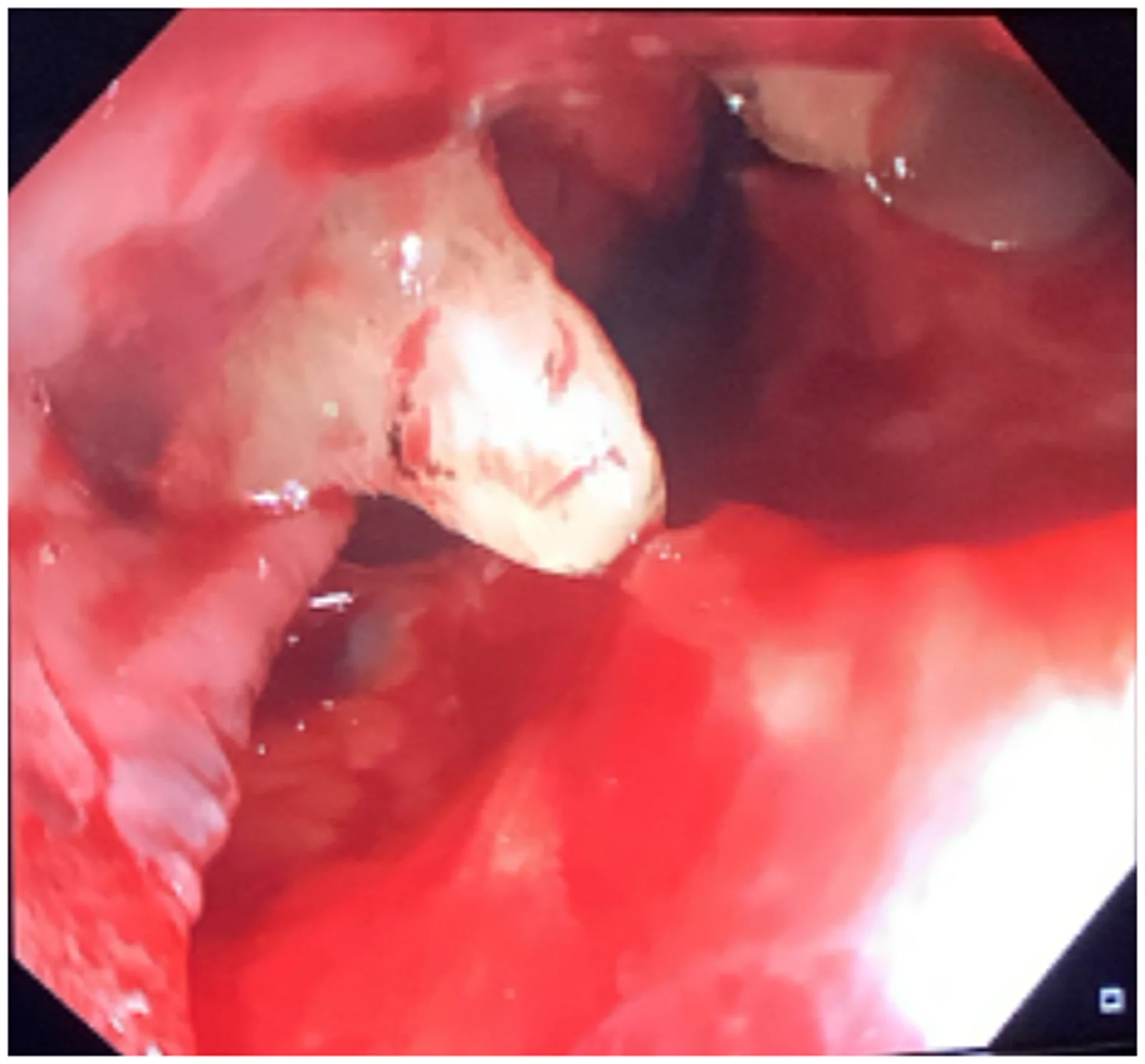
Intraoperative endoscopic image showing the impacted denture fragment lodged in the upper thoracic oesophagus.

Thoracotomy through the fifth intercostal space with partial resection of the fifth and sixth ribs revealed pneumomediastinum without any evidence for contamination or bleeding. Mobilization of the oesophagus revealed a 4 cm linear through-and-through perforation with localized inflammation, just superior to the azygos vein. The denture fragment was retrieved (Fig. 3). A T-tube was positioned across the defect. Mediastinal washout was performed, and apical and basal chest drains were inserted.

**Figure 3:**
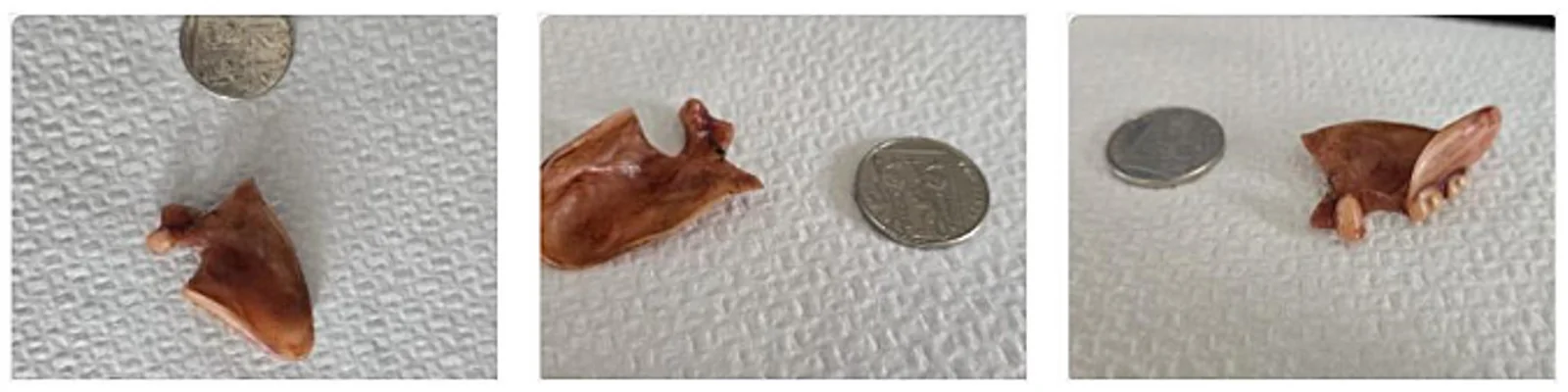
Retrieved denture fragment following surgical removal from the oesophagus. The fragment measures ~4 cm in length and demonstrates the irregular contours and sharp edges characteristic of dental prostheses. The denture fragment is placed next to a 20 pence Great British Pound (measuring 2.14 cm in diameter) for comparison.

Post-operatively, the patient remained intubated with ongoing oxygen requirements. Following extubation, persistent oxygen requirements prompted repeat imaging, which demonstrated mediastinal air and surgical emphysema. Broad-spectrum antimicrobial therapy was initiated, later escalated to voriconazole following Candida isolation on bronchoalveolar lavage. The post-operative course was complicated by suspected mediastinitis, candidiasis, and deconditioning, which were managed conservatively.

Nutritional management involved strict nil-by-mouth and total parenteral nutrition (TPN). Due to prolonged inability to tolerate oral intake, a nasojejunal (NJ) feeding tube was inserted on post-operative day (POD) 12 to facilitate enteral feeding. CT-TAP with oral contrast on POD 19 demonstrated no significant ongoing leak, and chest drains were removed. The patient was discharged with community support and outpatient follow-up for planned T-tube removal.

Seven weeks after surgery, oesophagogastroduodenoscopy (OGD) demonstrated a healthy oesophagus with the T-tube *in situ*. The T-tube was successfully removed endoscopically and subsequent CT-TAP confirmed no anastomotic leak.

## Discussion

OP carries significant morbidity and mortality, with outcomes heavily dependent on early recognition and prompt intervention. This case highlights the diagnostic challenges of radiolucent dentures, the importance of timely surgical decision-making following failed endoscopic retrieval, and the role of T-tube placement for complex thoracic perforations where primary repair may be unsafe.

Unlike radiopaque objects, such as coins or bones, dental prostheses are often poorly visualized on plain radiography, contributing to a reported misdiagnosis rate of up to 47% [8]. In our case report, the denture fragment was fortunately identified on the initial X-ray. Diagnostic oesophagoscopy is the preferred next step, with success rates exceeding 95% when performed early [9]. However, denture ingestion is associated with higher failure rates due to mucosal impaction, and when perforation is confirmed, continued endoscopy risks enlarging the defect and disseminating contamination [2, 4, 9–11]. Therefore, prompt surgical exploration is mandated [2, 10].

Primary repair remains the treatment of choice for perforations detected within 24 h, with a mortality of approximately 5.0% [12] to 9.5% [2]. In a case series of 43 thoracic perforations, when primary repair was attempted beyond 24 h, the complication rate rose to 61%, and increased further to 82% beyond 72 h [12]. The presence of localized inflammation and a 4 cm perforation with pneumomediastinum precluded safe primary closure, making T-tube placement a valuable alternative [2, 10, 12, 13]. The T-tube technique was first reported by Abbott *et al.* in 1970 [14] decompresses the oesophagus, diverts luminal contents externally, and allows a fibrous tract to form around the tube, which ultimately obliterates once removed [12, 13, 15].

In the same series of 43 thoracic perforations, delayed perforations managed with T-tube drainage had a mortality of only 8.7%, similar to the 5% mortality seen with early primary repair [12]. Although surgery was performed within 24 h in the present case, the decision to proceed with T-tube placement was based on the intraoperative findings of a large 4 cm perforation, localized mediastinal inflammation, and pneumomediastinum. These features have been associated with increased risk of anastomotic breakdown following primary closure [2, 10, 13]. A meta-analysis by Biancari *et al.* (2013) confirmed acceptable outcomes across a broader high-risk population, with a pooled mortality of 20% in T-tube repairs, reflecting the selection of the most complex cases for this technique [2]. The alternative of oesophagectomy carries a reported pooled mortality of 13.8% and is associated with permanent changes in patients’ quality of life [2].

This case highlights the diagnostic challenges posed by radiolucent dentures as a cause of OP, the importance of timely surgical escalation when endoscopic retrieval fails, and the value of T-tube drainage as an effective strategy for complex thoracic perforations where primary repair is unsafe. The favourable outcome in this high-risk patient supports the role of T-tube placement as a safe alternative to more radical resection, while reinforcing that a multidisciplinary approach is essential to optimize outcomes in this challenging patient population.
